# Author Correction: Identification of Genetically Important Individuals of the Rediscovered Floreana Galápagos Giant Tortoise (*Chelonoidis elephantopus*) Provides Founders for Species Restoration Program

**DOI:** 10.1038/s41598-018-22519-y

**Published:** 2018-03-12

**Authors:** Joshua M. Miller, Maud C. Quinzin, Nikos Poulakakis, James P. Gibbs, Luciano B. Beheregaray, Ryan C. Garrick, Michael A. Russello, Claudio Ciofi, Danielle L. Edwards, Elizabeth A. Hunter, Washington Tapia, Danny Rueda, Jorge Carrión, Andrés A. Valdivieso, Adalgisa Caccone

**Affiliations:** 1 Department of Ecology and Evolutionary Biology, Yale University, 165 Prospect St, New Haven, Connecticut 06520 United States of America; 2 Department of Biology, School of Sciences and Engineering, University of Crete, Vasilika Vouton, Gr-, 71300 Heraklio Crete, Greece; 3 Natural History Museum of Crete, School of Sciences and Engineering, University of Crete, Knossos Av., GR-71409 Heraklio Crete, Greece; 4 College of Environmental Science & Forestry, State University of New York, Syracuse, New York, 13210 United States of America; 5 Molecular Ecology Lab, School of Biological Sciences, Flinders University, GPO Box 2100, Adelaide, SA 5001 Australia; 6 Department of Biology, University of Mississippi, Oxford, Mississippi 38677 United States of America; 7 Department of Biology, University of British Columbia, Okanagan Campus, Kelowna, BC V1V 1V7 Canada; 8 Department Biology, University of Florence, 50019 Sesto Fiorentino (FI), Italy; 9 Life and Environmental Sciences, University of California, Merced, 5200 N Lake Rd, Merced California, 95343 United States of America; 10 Department of Natural Resources and Environmental Science, University of Nevada – Reno, Max Fleischmann Agricultural Building, Reno, NV 89557 USA; 11 Galapagos Conservancy, Fairfax, Virginia 22030 United States of America; 12 Galápagos National Park Directorate, Puerto Ayora, Galápagos Ecuador

Correction to: *Scientific Reports* 10.1038/s41598-017-11516-2, published online 13 September 2017

The original version of this Article contained an error in the title of the paper, where the word “Provides” was incorrectly given as “Provide”. This has now been corrected in the PDF and HTML versions of the Article and in the accompanying Supplementary Information file.

