# Supplementary material for: Identification of Genetically Important Individuals of the Rediscovered Floreana Galápagos Giant Tortoise (Chelonoidis elephantopus) Provides Founders for Species Restoration Program
Source: Sci Rep. 2017 Sep 13;7:11471. doi: 10.1038/s41598-017-11516-2 (PMC5597637; doi:10.1038/s41598-017-11516-2)
Supplement: Supplementary file 1 — Supplementary Materials [file 41598_2017_11516_MOESM1_ESM.pdf]

Supplementary Materials

Identification of Genetically Important Individuals of the Rediscovered Floreana Galápagos Giant Tortoise (Chelonoidis elephantopus) Provides Founders for Species Restoration Program

Joshua M. Miller[1†]*, Maud C. Quinzin[1†], Nikos Poulakakis[2,3], James P. Gibbs[4], Luciano B. Beheregaray[5], Ryan C. Garrick[6], Michael A. Russello[7], Claudio Ciofi[8], Danielle L. Edwards[9], Elizabeth A. Hunter[10], Washington Tapia[11,12], Danny Rueda[12], Jorge Carrión[12], Andrés A. Valdivieso[1], Adalgisa Caccone[1]*

1. Department of Ecology and Evolutionary Biology, Yale University, 21 Sachem St. New Haven, Connecticut, 06520, United States of America
2. Department of Biology, School of Sciences and Engineering, University of Crete, Vasilika Vouton, Gr-71300, Heraklio, Crete, Greece
3. Natural History Museum of Crete, School of Sciences and Engineering, University of Crete, Knossos Av., GR-71409, Heraklio, Crete, Greece
4. College of Environmental Science & Forestry, State University of New York, Syracuse, New York, 13210, United States of America
5. Molecular Ecology Lab, School of Biological Sciences, Flinders University, GPO Box 2100, Adelaide, SA, 5001, Australia
6. Department of Biology, University of Mississippi, Oxford, Mississippi, 38677, United States of America
7. Department of Biology, University of British Columbia, Okanagan Campus, Kelowna, BC V1V 1V7, Canada
8. Department Biology, University of Florence, 50019 Sesto Fiorentino (FI), Italy
9. Life and Environmental Sciences, University of California, Merced, 5200 N Lake Rd, Merced, California, 95343, United States of America
10. Department of Natural Resources and Environmental Science, University of Nevada – Reno, Max Fleischmann Agricultural Building, Reno NV 89557 USA
11. Galapagos Conservancy, Fairfax, Virginia, 22030, United States of America
12. Galápagos National Park Directorate, Puerto Ayora, Galápagos, Ecuador

*Corresponding authors: joshua.miller@yale.edu, adalgisa.caccone@yale.edu

†These authors contributed equally to this work

Lab Methods

We amplified ~700bp of the mitochondrial DNA control region using primers CytoR4 (5'-GCTTAACTAAAGCACCGGTCTTG-3')[1] and DLRev3 (5'-AATATTTGAGTTGTCGTGGG-3')[2]. Polymerase Chain Reactions (PCRs) were carried out in a volume of 25.0 µL using 5.0 µL of 5x GoTaq Flexi Buffer (Promega), 1.5 µL of $MgCl_2$ (25mM), 1.0 µL of each primer (10 µM), 1.5 µL of BSA (20 ng/µL; New England Biolabs), 0.1 µL GoTaq Flexi DNA Polymerase (5 U/µL; Promega), 11.9 µL water, and 1.0 µL of genomic DNA. Amplifications were performed using the following profile: initial denaturation at 95°C for 5min, 40 cycles of denaturation at 95°C for 30s, annealing at 50°C for 30s, and extension at 72°C for 45s, followed by a final extension at 72°C for 7min. PCR products were cleaned with AMPure Magnetic Beads and then sequenced with BigDye by Yale University's DNA Analysis Facility. Sequences were checked by eye in Geneious version 6.0.6[3] and then aligned to a reference database of 123 previously sequenced haplotypes representing all extant and extinct lineages.

We amplified 12 dinucleotide microsatellites in five multiplex co-amplifications. Primer sequences and combinations are presented in Table S1. Multiplexes 1 - 4 were conducted in 15.0 µL volumes while multiplex 5 was conducted in 10.0 µL volumes. Each PCR contained 5.0 µL Qiagen Type-it Multiplex PCR Master Mix (2x), 0.17µL each primer (10µM), 1.0 µL genomic DNA, and water to volume. The thermocycler profile for multiplexes 1 - 4 was: initial denaturation at 95°C for 5min, 10 touchdown cycles with a 1°C reduction in annealing temperate per cycle 95°C 25s, 63 – 54°C 1min, 72°C 30s, followed by 35 cycles of 95°C 25s, 53°C 20s, 72°C 30s, and a final extension at 72°C for 15min. The thermocycler profile for multiplex 5 was: initial denaturation at 95°C for 5min, 30 cycles of denaturation at 95°C for 25s, annealing at 53°C for 20s, and extension at 72°C for 30s, with a final extension at 60°C for 30min. Allele calls were checked and recorded as raw sizes from GeneMapper 5 (Applied BioSystems). New genotypes were added to a reference database containing 277 individuals representing all extant and extinct lineages, a subset of those used in previous studies[4,5]. Raw alleles sizes were then binned using Tandem 2[6] for consistency across datasets.

Ancestry Assignment

To establish the number of genotypic clusters (K) in the archipelago we initially ran the Bayesian clustering program STRUCTURE version 2.3.4[7,8] on a reference dataset of 277

individuals, which includes all extant and extinct lineages. Specifically, varying K from 1 – 15 we ran 20 iterations for each K assuming admixture, correlated allele frequencies among populations, and no prior location information. Runs included 500,000 Markov Chain Monte Carlo (MCMC) iterations following 100,000 burn-in iterations. We then used CLUMPAK[9] for post-processing outputs and for determining the optimal K which we defined as the K for which median Ln $\Pr(X|K)$ value was maximized. When assigning ancestry to the 150 newly collected samples we restricted the reference samples to lineages previously found to be on Volcano Wolf[10]: those from Española, San Cristóbal, Central Isabela (La Cazuela, Volcano Alcedo, and Volcano Darwin), Floreana, Pinta, Piedras Blancas (PBL), and Puerto Bravo (PBR) for a total of 155 samples. We then ran $K = 7$ leaving all other parameters the same as above.

Following this initial assessment, we further quantified the genetic ancestry of the newly collected individuals using additional assignment approaches and expanding the reference database to include simulated genotypes. The simulated genotypes were obtained using HybridLab[11] and corresponded to crosses amongst specific reference populations. HybridLab generates multi-locus genotypes for offspring of two specified parental populations based on empirical allele frequencies in each population[11]. Genotypes were generated from: 1) mating within the genetic lineages from Floreana, Española, PBL, and PBR; 2) hybridization amongst the four parental populations (F1's); 3) mating between F1's (F2's); and 4) backcrosses of F1's to the parental lineages (BC's). One hundred individuals were simulated from each pairing. Q-values from STRUCTURE for the simulated hybrids (see below) were in the range expected for each "class" of hybrid (Supplementary Figure S4) indicating we have the ability to identify individuals with Floreana ancestry across various levels of admixture.

The simulated genotypes were then added to the full reference dataset (for a total of 6210 genotypes) for assignment tests using GeneClass2[12]. Here the 150 new samples were assigned to one of the reference populations using the method of Paetkau et al.[13]. Individuals were considered to be potential hybrids if their "relative score" to ancestry categories involving Floreana were greater than 65%.

Potential Floreana hybrids identified by GeneClass2 were further examined in NEWHYBRIDS[14]. NEWHYBRIDS can only consider two parental populations at a time, so we restricted analyses to the following pairwise combinations of parental populations: 1) Española with Floreana, 2) Española with PBR, 3) Floreana with PBR, 4) Floreana with PBL, and 5) PBL

with PBR. For each pair, NEWHYBRIDS was run using 100,000 burn-in and more than 300,000 iterations post burn-in. Allele frequencies for the ancestry matrix were calculated from the simulated genotypes. For each individual, posterior probability values assigned to each hybrid class (F1, F2, etc.) and an individual as belonging to a specific group if the sum of the posterior probability for that group was >0.70.

In addition, we re-ran STRUCTURE using the simulated crosses as the reference populations. Here, we conducted two analyses to avoid overloading the dataset with reference samples relative to the new ones we wished to assign ancestry[15,16]. The first run considered crosses among Floreana, Española, and PBR whereas the second run considered crosses among Floreana, PBL, and PBR. Individuals from the 2015 expedition were included in a run only if they had assignment from GeneClass2 to Floreana and Española in the first run, and Floreana but not Española in the second run. For both analyses K was set to 3.

Finally, we also assigned the potential hybrids using discriminant analysis of principal components (DAPC), as implemented in R package *adegenet*[17,18]. DAPC was chosen as it does not make some of the underlying assumptions that other methods such as STRUCTURE do (e.g., populations are in Hardy-Weinberg equilibrium, markers are in linkage equilibrium). This method provides discrimination of individuals into pre-defined groups[19], in our case known F_1's, F_2's, etc. Briefly, DAPC works by first transforming the multivariate genetic data with principal component analysis (PCA) to reduce its complexity, and then using discriminant functions to partition variation such that the between-group variation is maximized while the within-group variation is minimized. Once the number of principal components (PCs) and discriminant functions were determined for the reference samples, the putative hybrids were projected onto these results using the centering, scaling, and discriminant functions of the reference samples. We used cross-validation (with 50 repetitions) to determine the optimal number of PCs to retain in the reference sample. Assignments were made in three groups to focus on identifying potential hybrids involving Floreana ancestry: 1) Floreana with Española, 2) Floreana with PBL, and 3) Floreana with PBR.

To examine the precision and accuracy of our ancestry assignments we conducted additional analyses in STRUCTURE. First, we implemented a jackknifing procedure for the loci in our dataset, sequentially removing one locus and re-running STRUCTURE. These analyses considered the dataset comprised of the 7 reference populations expected to be on VW and used

the following run parameters: admixture, correlated allele frequencies, 100,000 burn-in iterations, and 500,000 MCMC iterations. For each 11-locus data-subset we ran 20 repetitions of K=7 and the results were post-processed with CLUMPAK. For each major cluster identified by CLUMPAK q-values to the FLO cluster were then compared. Second, we ran STRUCTURE using all 12 loci along with ANCESTDIST option, which collects information about the distribution of q-values for each individual. We used 90% intervals (the default setting) leaving all other run parameters the same as above. For these analyses we considered two separate datasets: 1) the 7 reference populations expected to be on VW; setting K=7. 2) the 4 reference populations examined in the GeneClass and NEWHYBRIDS analyses (ESP, FLO, PBL, PBR) along with simulated hybrid individuals (1000 each for simulated parental, F1s, and BCs); setting K=4. For each dataset, we calculated individual average q-values and CIs in the FLO cluster across the 20 runs.

Supplementary References

1. Caccone, A., Gibbs, J. P., Ketmaier, V., Suatoni, E. & Powell, J. R. Origin and evolutionary relationships of giant Galapagos tortoises. *Proc. Natl. Acad. Sci. U. S. A.* **96,** 13223–13228 (1999).
2. Beheregaray, L. B., Ciofi, C., Caccone, A., Gibbs, J. P. & Powell, J. R. Genetic divergence, phylogeography and conservation units of giant tortoises from Santa Cruz and Pinzon, Galapagos Islands. *Conserv. Genet.* **4,** 31–46 (2003).
3. Kearse, M. *et al.* Geneious Basic: An integrated and extendable desktop software platform for the organization and analysis of sequence data. *Bioinformatics* **28,** 1647–1649 (2012).
4. Garrick, R. C. *et al.* Genetic rediscovery of an 'extinct' Galapagos giant tortoise species. *Curr. Biol.* **22,** R10–R11 (2012).
5. Edwards, D. L. *et al.* The genetic legacy of Lonesome George survives: Giant tortoises with Pinta Island ancestry identified in Galapagos. *Biol. Conserv.* **157,** 225–228 (2013).
6. Matschiner, M. & Salzburger, W. TANDEM: Integrating automated allele binning into genetics and genomics workflows. *Bioinformatics* **25,** 1982–1983 (2009).
7. Falush, D., Stephens, M. & Pritchard, J. K. Inference of population structure using multilocus genotype data: Linked loci and correlated allele frequencies. *Genetics* **164,** 1567–1587 (2003).

8. Pritchard, J. K., Stephens, M. & Donnelly, P. Inference of population structure using multilocus genotype data. *Genetics* **155,** 945–959 (2000).

9. Kopelman, N. M., Mayzel, J., Jakobsson, M., Rosenberg, N. A. & Mayrose, I. Clumpak: a program for identifying clustering modes and packaging population structure inferences across K. *Mol. Ecol. Resour.* **15,** 1179–1191 (2015).

10. Poulakakis, N., Russello, M., Geist, D. & Caccone, A. Unravelling the peculiarities of island life: vicariance, dispersal and the diversification of the extinct and extant giant Galapagos tortoises. *Mol. Ecol.* **21,** 160–173 (2012).

11. Nielsen, E. E., Bach, L. A. & Kotlicki, P. HYBRIDLAB (version 1.0): a program for generating simulated hybrids from population samples. *Mol. Ecol. Notes* **6,** 971–973 (2006).

12. Piry, S. *et al.* GENECLASS2: A Software for Genetic Assignment and First-Generation Migrant Detection. *J. Hered.* **95,** 536–539 (2004).

13. Paetkau, D., Waits, L. P., Clarkson, P. L., Craighead, L. & Strobeck, C. An Empirical Evaluation of Genetic Distance Statistics Using Microsatellite Data From Bear (Ursidae) Populations. *Genetics* **147,** 1943 LP-1957 (1997).

14. Anderson, E. C. & Thompson, E. A. A model-based method for identifying species hybrids using multilocus genetic data. *Genetics* **160,** 1217–1229 (2002).

15. Wang, J. The computer program Structure for assigning individuals to populations: easy to use but easier to misuse. *Mol. Ecol. Resour.* doi:10.1111/1755-0998.12650

16. Puechmaille, S. J. The program STRUCTURE does not reliably recover the correct population structure when sampling is uneven: sub-sampling and new estimators alleviate the problem. *Mol. Ecol. Resour.* **16,** 608–627 (2016).

17. Jombart, T. & Ahmed, I. adegenet 1.3-1: new tools for the analysis of genome-wide SNP data. *Bioinformatics* **27,** 3070–3071 (2011).

18. Jombart, T. adegenet: a R package for the multivariate analysis of genetic markers. *Bioinformatics* **24,** 1403–1405 (2008).

19. Jombart, T., Devillard, S. & Balloux, F. Discriminant analysis of principal components: a new method for the analysis of genetically structured populations. *BMC Genet.* **11,** doi:10.1186/1471-2156-11-94 (2010).

20. Ciofi, C., Milinkovitch, M. C., Gibbs, J. P., Caccone, A. & Powell, J. R. Microsatellite

analysis of genetic divergence among populations of giant Galapagos tortoises. *Mol. Ecol.* **11,** 2265–2283 (2002).

21. Ciofi, C. *et al.* Phylogeographic history and gene flow among giant Galapagos tortoises on southern Isabela Island. *Genetics* **172,** 1727–1744 (2006).
22. Milinkovitch, M. C. *et al.* Genetic analysis of a successful repatriation programme: giant Galapagos tortoises. *Proc. R. Soc. B-Biological Sci.* **271,** 341–345 (2004).
23. Queller, D. C. & Goodnight, K. F. Estimating relatedness using genetic-markers. *Evolution (N. Y).* **43,** 258–275 (1989).
24. Gonçalves da Silva, A. & Russello, M. A. iRel: software for implementing pairwise relatedness estimators and evaluating their performance. *Conserv. Genet. Resour.* **3,** 69–71 (2011).

Table S1: Primer sequences and multiplex mixes for 12 dinucleotide microsatellites used in this study

Primer Name	Primer Sequence 5'-3'	Label	Multiplex	Citation
**Gal45 _F**	TATCTCCTTCCACACGGAGATGGG	NED	1	[20]
**Gal45_R**	GTTTCCCCAAAGTAAAGTTAGCTCTCTCA			
**Gal50_F**	TGGGACAGGCAAACTAACAAAACTT	FAM	2	[20]
**Gal50_R**	GTTTTGCAGAAGTTAATCCCTTTCTCCTT			
**Gal75_F**	GAAGCCATTTACCACAAACTTATT	FAM	1	[20]
**Gal75_R**	GTTTGTTACCATAGCATTCCTGATTATAG			
**Gal94_F**	CTTCTATTTCCCAACCATCT	HEX	3	[20]
**Gal94_R**	GTTTAACTTTATATTTGTGTGCATATT			
**Gal100_F**	TCTTAATAAATTCCATGAGTTGAGCT	FAM	3	[20]
**Gal100_R**	GTTTAGGGTGATTTCATAAACAAACAGAA			
**Gal127_F**	TAACTATAAACATCAACTGGCAGAA	HEX	4	[20]
**Gal127_R**	GTTTAGTGTCATCTGCATATGC			
**Gal136_F**	ATGAGATGTATGTACAGAAAATATA	FAM	4	[20]
**Gal136_R**	GTTTCTGGAGGGAAGTAAGAATC			
**Gal159_F**	AATATTTGAAGATACTCATCCTCGA	NED	2	[20]
**Gal159_R**	GTTTTTATGTGCTTGTGTCATCTTTTT			
**Gal263_F**	GGGAAAGTACTATTTCCAGAGCTGG	HEX	1	[20]
**Gal263_R**	GTTTGCTGAGGCTAGCTAATTTTTATGT			
**GAL194_F**	ATGTAGTTGCATCATCCCTGGAGCA	FAM	5	[21]
**GAL194_R**	AATAACGACTGGAATTGGCAATGG			
**GAL288_F**	AGGCTCAGTGCCATCAGAGGTATG	HEX	5	[21]
**GAL288_R**	ATCCAGACCCCACATCCTGCTATT			
**AC063_F**	GTTTCTTGGGGAGGGGGCTGAATCTTGAT		5	[22]
**AC063_R**	CTGGAAGCAGGAGACAAAAGGGAG	56-TAMN		

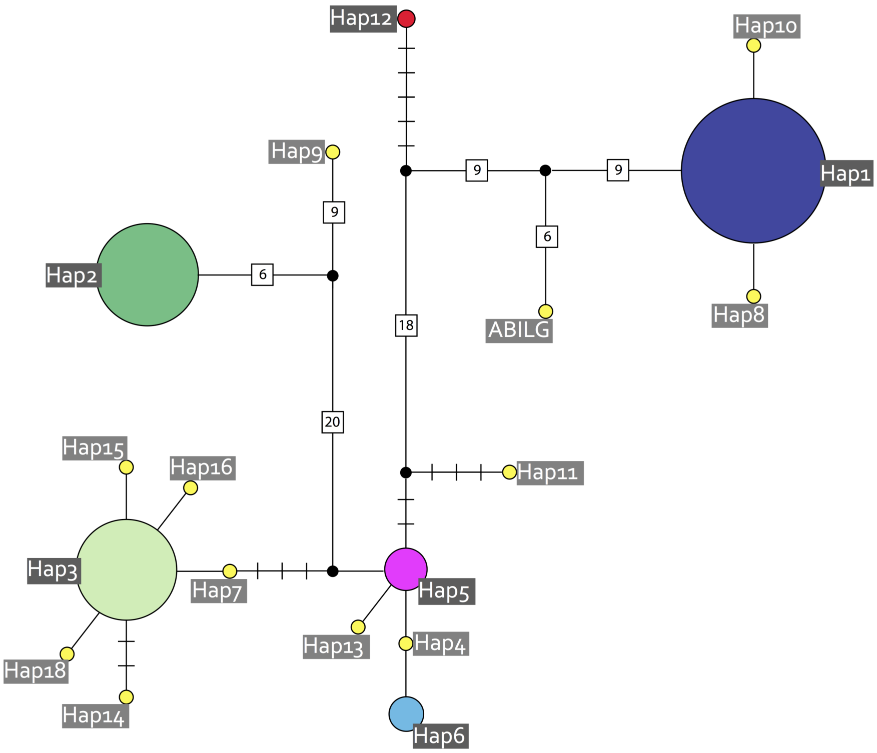

Fig S1: Mitochondrial DNA haplotype network for all 150 samples assessed in this study. Colored circles represent haplotypes observed in the current study with the size of the circle proportional to the number of individuals with that haplotype. Haplotypes are from Floreana (dark green), Española (purple), shared between Santiago and Volcano Wolf (light green, pink and teal), or shared between San Cristobal and Santa Cruz (red). Yellow circles represent haplotypes observed on Volcano Wolf from a survey of 1600 tortoises in 2008[4], but not seen in the current study. Black circles represent hypothetical haplotypes that were not sampled or are extinct. Where the number of mutation events haplotypes is greater than 1 each event is represented by a short line crossing the lines linking each haplotype until 5 events after which the number is listed in the box between haplotypes. Haplotype names correspond to those used in [4].

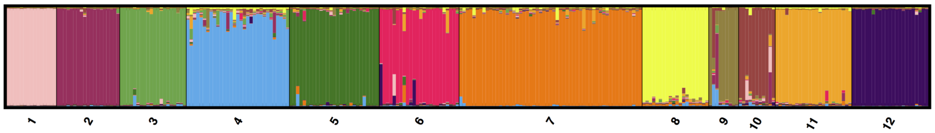

Fig S2: STRUCTURE bar plot for the 255 archipelago-wide reference samples. Each individual is represented as a vertical bar, with colors denoting the different genetic lineages. The proportion of color in a bar is equal to the ancestry (Q-value) to a given cluster. Numbers below the figure correspond to 1) Española, 2) San Cristóbal, 3) Cerro Fatal (Santa Cruz), 4) La Caseta (Santa Cruz), 5) Pinzon 6) Santiago, 7) Southern Isabela, 8) Central Isabela, 9) Pinta, 10) Floreana, 11) PBL (Northern Isabela, V. Wolf), 12) PBR (Northern Isabela, Volcano Wolf).

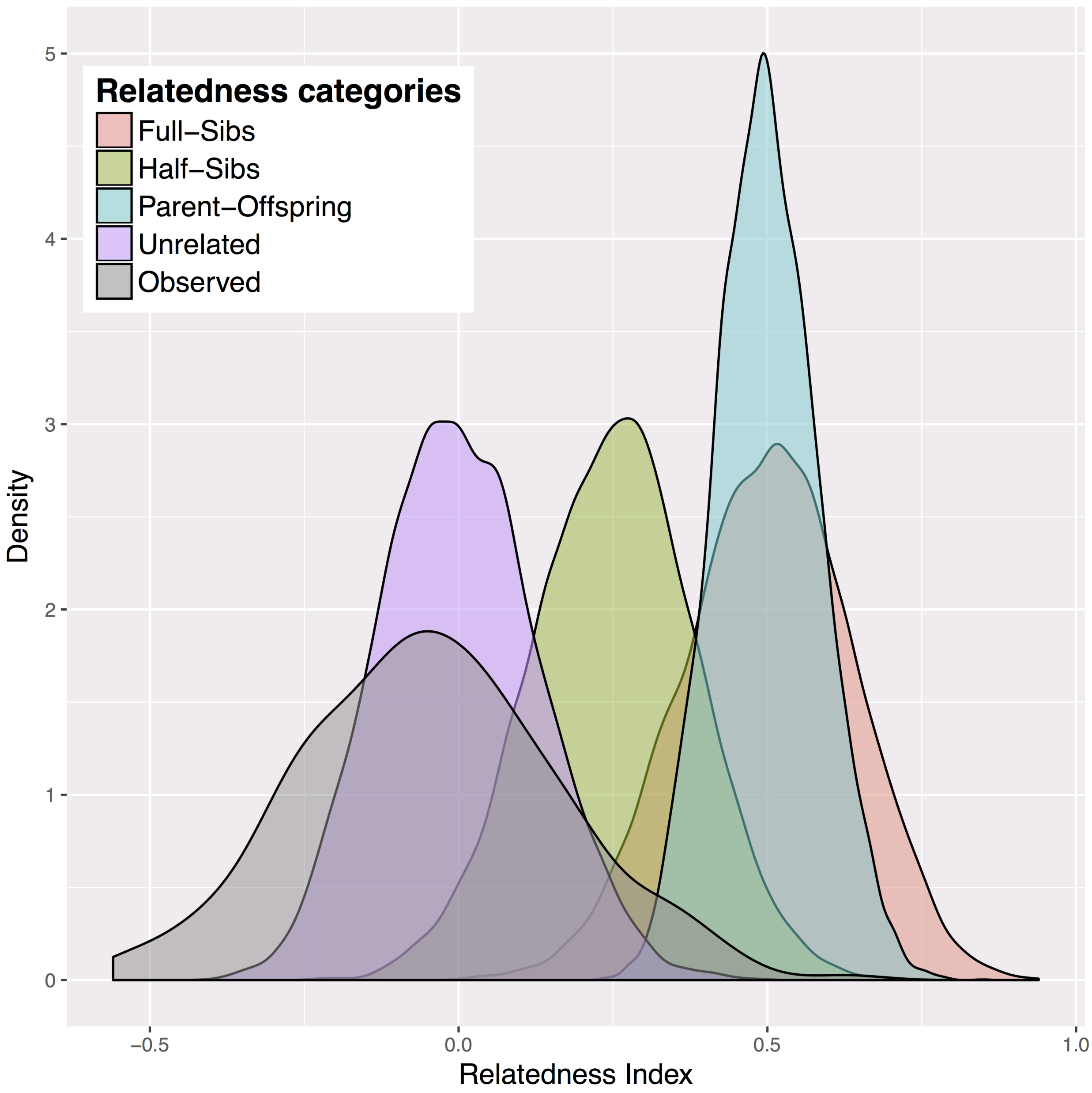

Fig S3: Plot of the frequency distribution of pairwise relatedness estimates for simulated relatedness categories and the observed distribution from the 23 giant Galapagos tortoises currently in the breeding center on Santa Cruz Island. Estimates were made using 10,000 dyads and the metric of Queller and Goodnight[23] as implemented in the program iRel[24].

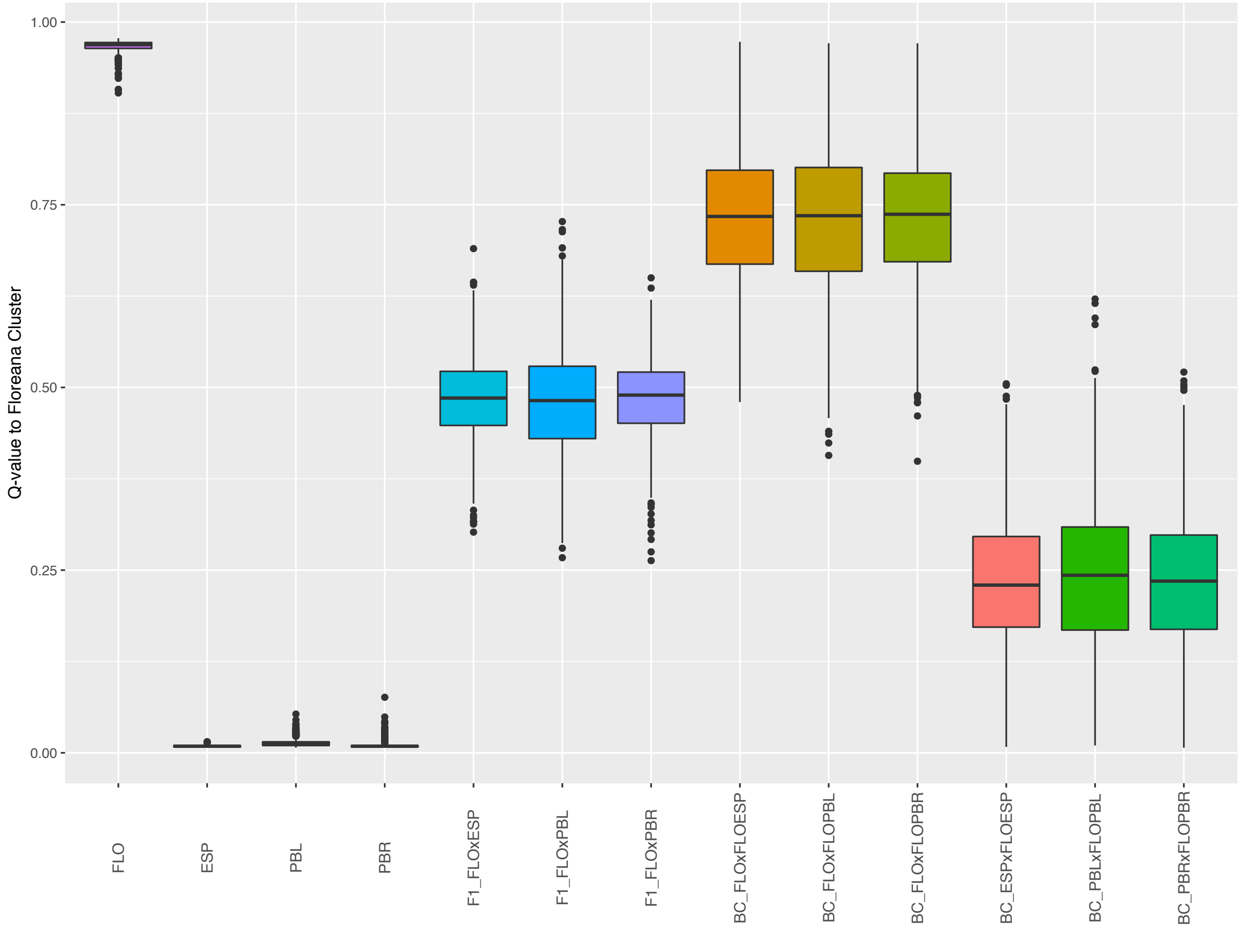

Fig S7: Boxplots showing the distribution of Q-values to the Floreana genetic cluster for different simulated hybrid classes. Each boxplot is based on 1000 simulated individuals and the reference samples, where applicable. Abbreviations are: BC = backcross, F_1 = first generation mating, ESP = Española, and FLO = Floreana.

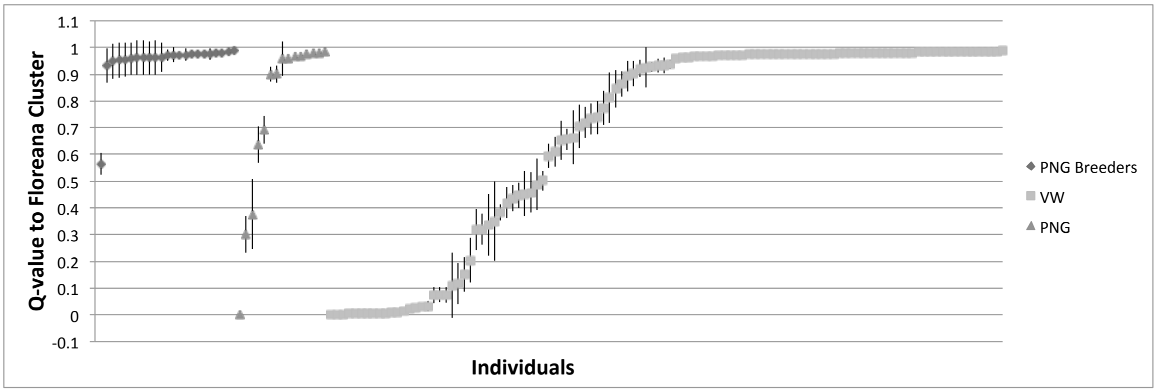

Fig S5: Average Q-values ± SD to the Floreana cluster after jackknifing the dataset, serially removing one locus and rerunning STRUCTURE. Depicted are the 150 individuals collected in 2015. For this figure the STRUCTURE runs included individuals from 7 reference populations from the species from Española, Floreana, Northern Isabela (V. Wolf, PBL and PBR), San Cristobal, Pinta, and Central Isabela). VW = individuals remaining on Volcano Wolf. PNG = individuals currently in the breeding center. Black squares represent the 23 individuals considered for the captive breeding program.

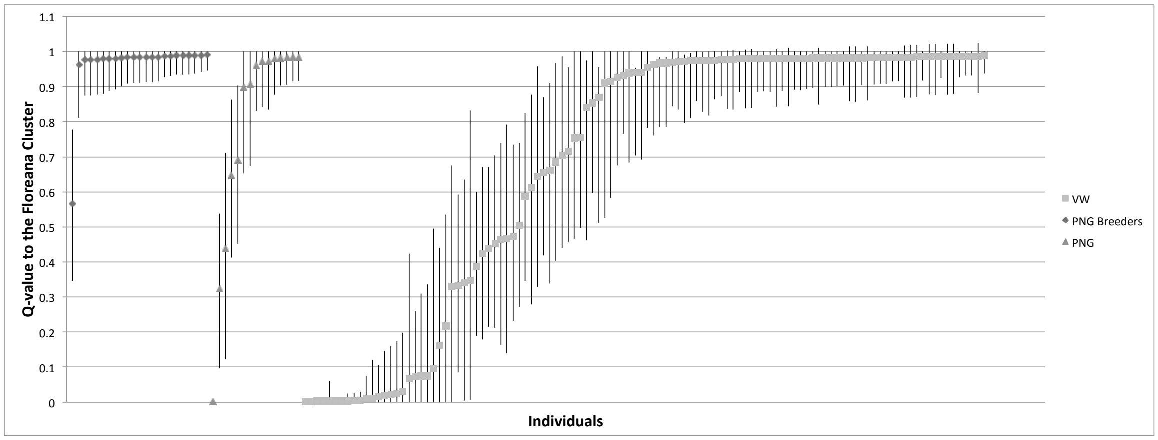

Fig S6: Individual Q-values and 90% distributions to the FLO cluster. Depicted are the 150 individuals collected in 2015. For this figure the STRUCTURE run included individuals from 7 reference populations from the 6 species from Española, Floreana, Northern Isabela (V. Wolf: PBL and PBR), San Cristobal, Pinta, and Central Isabela). VW = individuals remaining on Volcano Wolf. PNG Breeders = individuals currently in the breeding center considered for the captive breeding program. PNG = individuals currently in the breeding center not considered for the captive breeding program.

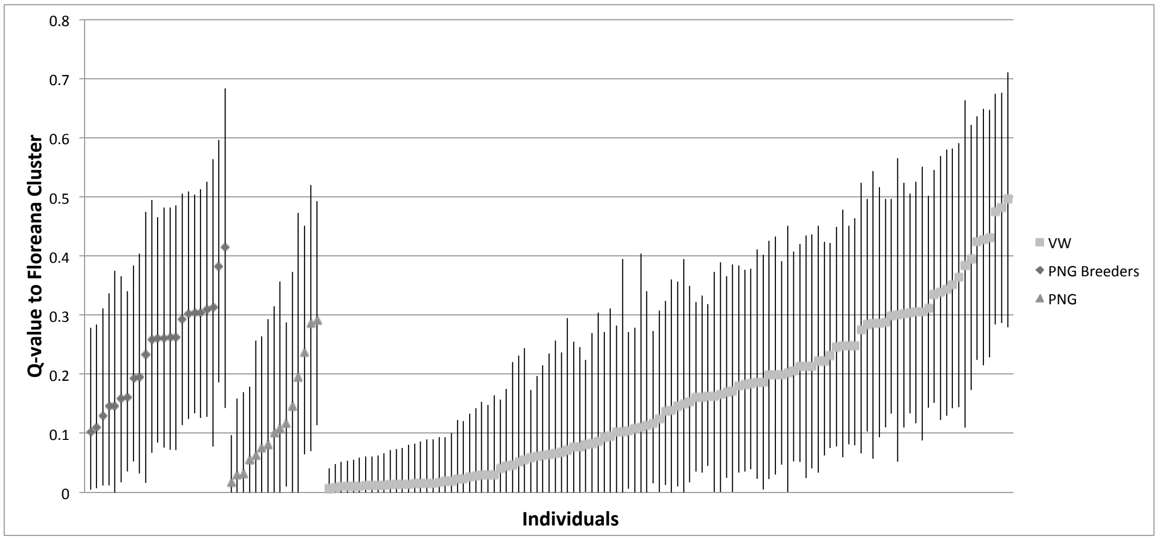

Fig S7: Individual Q-values and 90% distributions to the FLO cluster. Depicted are the 150 individuals collected in 2015. For this figure the STRUCTURE run included individuals from 4 reference populations from the 3 species form Española, Floreana, and Northern Isabela (V. Wolf: PBL and PBR) along with simulated hybrid. VW = individuals remaining on Volcano Wolf. PNG Breeders = individuals currently in the breeding center considered for the captive breeding program. PNG = individuals currently in the breeding center not considered for the captive breeding program.
